# Plaster-Of-Paris

**Published:** 1872-12

**Authors:** 


### ARTICLE VI.

### *Plaster-of-Paris.*

The quarrying of gypsum and the manufacture of plaster are important industries in Paris, and we have recently taken the opportunity of visiting one of the establishments of this kind, the best arranged—that of M. Morel, at Montreuil. The plaster-of-Paris, or gypsum, consists, as is well known, of hydrated sulphate of lime. The water being removed, the stone is ground into powder. When this is afterwards mixed with water it combines itself again, and forms a solid mass, which is employed in an infinite variety of ways. The abundance of gypsum at Montmartre, Pantin, Menilmontant, Belleville, Charonne, Montreuil, etc., all close to Paris, even within the city limits, the good quality of, and the large demand for the plaster, and the ease with which it is employed, have caused the development of this great industry in the capital. The plaster of Paris has a European, and even a still more extended reputation. It is employed everywhere, and is put to the most varied uses. It is moulded into hollow bricks, and tubular blocks, in building up partitions and walls, for paving slabs, and for smoke conduits to chimneys. One sees, even in the neighborhood of the quarries, houses of three and four stories, which are built in moulded stones of plaster, or made in plaster in such a manner that they form a monolith.

The bed of gypsum worked at Pantin is horizontal; it has a thickness of 37 feet, 2 inches. There is also a small bed adjacent, and of little thickness, but this is not quarried as a rule. The gypsum of this bed is almost entirely crystalized, and there are found there, in abundance, those beautiful specimens called *fers de lance*, on account of their form. These fragments split with ease into thin transparent leaves, and when the apparent limit of divisibility has been found with the blade of a knife, if one takes one of the leaves, which has less than 1-80th of an inch of thickness, and heats it, it exfoliates into more than twenty films, as the water it contains is heated and disengages itself into steam.

The bed of gypsum that is excavated is covered by some 40 feet of earth, consisting of calcareous deposits, and marl, and clay. It is excavated for the most part, by subterranean galleries, but it is sometimes found to be more economical to work from the surface, in spite of the great thickness of superincumbent earth, because there are numerous situations where the excavated material employed to fill elsewhere can be made a source of revenue, while the limestone can be sold to make lime, and the clay to make earthenware, or bricks.

It is thus that the quarry of Eprisette, worked at first in galleries by M. Morel, is changed at the present time into open excavations.

The gypsum is extracted by blasting. Holes are pierced in the rock, which, for the most part, is sufficiently soft for a workman to drive in less than an hour a hole from 4 ft. 6 in., to 6 ft. deep, and 2 ft. in diameter. After a blast, the rock is struck with crowbars, which divides it into blocks from 30 to 40 metres cube, advantage being taken of the numerous faults in the material, which the workmen learn to recognize at a glance, and which they call "*maillances.*" A heavy blow, or the introduction of a pick, at the right spot, divides easily the largest rocks into convenient fragments.

These fragments are loaded upon trolleys, which follow the face of the gallery, or cutting, or tramways, and which lead up to the eight furnaces composing the factory. These kilns, or furnaces, are of the simplest form. They consist of an end wall 15 ft. high, and the square hearth that they surround carries perpendicularly to the end wall five gratings, through which passes the air necessary for combustion. On the ground, the largest blocks of gypsum are arranged in such a manner as to construct, above these gratings, arches sufficiently high to receive the fuel for burning the material. The spaces intervening are filled up with other fragments of rock, more of which is added from above, so that the height of the mass is raised. When the greatest height conveniently attainable by hand is reached, the charging of the kiln is continued from trolleys brought upon inclined planes, which are also supplied with rails. This is carried on until the height of the charge is equal to that of the walls of the kiln. All the interstices are then carefully packed with small fragments of the stone, and the front of the furnace, which is raised by a low wall, receives a movable cover of plate iron, intended to prevent the loss of heat by radiation; and to retain such morsels of stone as become detached during the operation of baking, the joints in front of the kiln are luted.

Everything being then prepared, fagots are placed within the arches and lighted, and when the embers are in full glow and the arches half empty, they are charged with *briquettes* of artificial fuel, and the fire is so managed, by regulating the access of air, that the baking of the mass is effected equally throughout without any extremes of excessive or imperfect burning. The operation is complete in 24 hours.

The employment of *briquettes* is one of the improvements introduced by M. Morel into his establishment. The baking was generally done with wood, and the substitution of coal has effected a saving of two-thirds of the total quantity produced. There is a comparatively small loss of heat in this apparatus, so simple and apparently so primitive. In calculating the calorific power of the quantity of fuel consumed and the amount of heat hecessary to evaporate all the water contained in the gypsum, it is found that he utilizes one half of the available heat, which is certainly a satisfactory result, considering all the various losses inseparable from an incidental enterprise.

After the calcination is complete, the furnace is allowed to cool, and the burnt gypsum is again loaded into wagons and carried off the tramway to the grinding mills. This part of the manufacture consists of two parts. There are mill stones in cast-iron or stone, banded with rings of iron and turning in a circular trough with a grated bottom. The calcined stone is fed into the mill, and those parts which are ground down extremely fine pass through its gratings. The rest is removed by a suitable mechanical appliance for grinding.

One of the mills carries a most ingenious arrangement for screening the fine powder. Below the grate there is a strainer in the form of a truncated cone. Of the powder which falls upon this strainer through the base of the annular grate, part passes through the meshes and escapes through the lower part of the apparatus; the rest slides on the conical strainer, falling on a table at the bottom, and is constantly lifted by a chain and replaced on the table of the mill. After the powder is sufficiently ground, it is conveyed below into a storehouse, where it is placed in bags. The machines are driven by a 12-horse steam engine.

The whole of this establishment is ably arranged and managed, from the quarries to the plaster depot; and the working out of all the practical details does honor to the able proprietor who created them, and who still works daily to improve them.—*Engineering.*

